# Diagnostic efficacy and clinical utility of whole-exome sequencing in Czech pediatric patients with rare and undiagnosed diseases

**DOI:** 10.1038/s41598-024-79872-4

**Published:** 2024-11-20

**Authors:** Katerina Slaba, Petra Pokorna, Robin Jugas, Hana Palova, Dagmar Prochazkova, Stefania Aulicka, Klara Spanelova, Pavlina Danhofer, Ondrej Horak, Jana Tuckova, Petra Kleiblova, Renata Gaillyova, Matej Hrunka, Martin Jouza, Blanka Pinkova, Jan Papez, Petra Konecna, Jana Zidkova, Petr Stourac, Jaroslav Sterba, Regina Demlova, Eva Demlova, Petr Jabandziev, Ondrej Slaby

**Affiliations:** 1 Department of Pediatrics, University Hospital Brno, Faculty of Medicine, Masaryk University, Cernopolni 9, 613 00 Brno, Czech Republic; 2 Central European Institute of Technology, Masaryk University, Brno, Czech Republic; 3 Department of Biology, Faculty of Medicine, Masaryk University, Kamenice 5, 625 00 Brno, Czech Republic; 4 Department of Pediatric Neurology, University Hospital Brno, Faculty of Medicine, Masaryk University, Brno, Czech Republic; 5 Institute of Medical Biochemistry and Laboratory Diagnostics, First Faculty of Medicine, Charles University and General University Hospital in Prague, Prague, Czech Republic; 6 Institute of Biology and Medical Genetics, First Faculty of Medicine, Charles University and General University Hospital in Prague, Prague, Czech Republic; 7 Department of Medical Genetics and Genomics, University Hospital Brno, Faculty of Medicine, Masaryk University Brno, Brno, Czech Republic; 8 Centre of Molecular Biology and Genetics, Department of Hematology, Oncology and Internal Medicine, Masaryk University and University Hospital Brno, Brno, Czech Republic; 9 Department of Pediatric Anesthesiology and Intensive Care Medicine, University Hospital Brno and Faculty of Medicine, Masaryk University, Brno, Czech Republic; 10 Department of Simulation Medicine, Faculty of Medicine, Masaryk University, Brno, Czech Republic; 11 Department of Pediatric Oncology, Faculty of Medicine, University Hospital Brno, Masaryk University, Brno, Czech Republic; 12 Department of Pharmacology/CZECRIN, Masaryk University Faculty of Medicine, Brno, Czech Republic; 13 Center of Precision Medicine, Department of Pathology, University Hospital Brno, Faculty of Medicine, Masaryk University, Brno, Czech Republic

**Keywords:** Rare genetic diseases, Undiagnosed patients, Whole-exome sequencing, Genetics research, Paediatric research

## Abstract

In the last decade, undiagnosed disease programs have emerged to address the significant number of individuals with suspected but undiagnosed rare genetic diseases. In our single-center study, we have launched a pilot program for pediatric patients with undiagnosed diseases in the second-largest university hospital in the Czech Republic. This study was prospectively conducted at the Department of Pediatrics at University Hospital Brno between 2020 and 2023. A total of 58 Czech patients with undiagnosed diseases were enrolled in the study. All children underwent singleton WES with targeted phenotype-driven analysis. We identified 28 variants, including 11 pathogenic, 13 likely pathogenic, and 4 VUS according to ACMG guidelines, as diagnostic of genetic diseases in 25 patients, resulting in an overall diagnostic yield of 43%. Eleven variants were novel and had not been previously reported in any public database. The overall clinical utility (actionability) enabling at least one type of change in the medical care of the patient was 76%, whereas the average number of clinical implications to individual patient care was two. Singleton WES facilitated the diagnostic process in the Czech undiagnosed pediatric population. We believe it is an effective approach to enable appropriate counseling, surveillance, and personalized clinical management.

## Introduction

Rare diseases (RD) are clinically heterogeneous, predominantly hereditary, multisystemic diseases with a very low incidence (prevalence) in the general population, which impact on the quality of life and social inclusion of patients or threaten their lives. A disease is defined as rare in the European Union if it affects less than one patient per 2000 individuals. The severity of RD is that there are more than 8000 different RDs, so the cumulative number of patients is not negligible, estimated to be 1 in 16 people in the general population, despite the low prevalence of individual clinical entities^1^.

Because a large proportion of rare diseases have a genetic basis, obtaining an accurate molecular diagnosis is crucial for appropriate clinical management, family, and reproductive counseling and support. However, it has been estimated that at least half of those with rare genetic diseases remain undiagnosed despite ‘standard’ clinical care, and these patients then undergo a so-called “diagnostic odyssey”^1,2^.

Over the last 15 years, Undiagnosed Diseases Programs have emerged to address the significant number of individuals with suspected but undiagnosed rare genetic diseases, integrating research and clinical care to optimize diagnostic outcomes. The first formal Undiagnosed Diseases Program was established in 2008 by the National Institutes of Health (NIH) in the United States, followed by the creation of the NIH-funded Undiagnosed Disease Network (UDN) in 2014^2,3^. Analogous initiatives, programs, and networks for undiagnosed diseases were subsequently created in Canada^4^, Great Britain^5^, Japan^6^, Spain^7^, Italy^8^, and other countries (for review^1^). In the Czech Republic, no such program for patients with undiagnosed diseases is formally established currently.

Along with developing these specialized programs for patients with undiagnosed diseases, the last decade has seen a rapid shift towards using whole-exome sequencing (WES) and whole-genome sequencing (WGS) as the first-line diagnostic test for patients with suspected genetic disease. However, many physicians still often consider it a last resort. The growing number of WES studies improved diagnostic rate by allowing concomitant examination of genes more comprehensively compared with conventional genetic tests, such as gene panels and chromosomal microarray analysis (CMA)^9–11^. In a recent meta-analysis from 2023, the median diagnostic yield in 13 WES studies on patients with rare diseases was 43%, and the median clinical utility, available in only four studies, was 44%.^12^ In addition, the clinical utility of these studies increased by approximately 3% year-on-year, and from a cost-utility perspective, the study concluded that clinical management changes due to WES were cost-saving^12^. This meta-analysis only further confirmed previous data that clearly justified using WES as an essential diagnostic method in patients with rare diseases. Recently, it has been shown that WGS overcomes some of the limitations of WES and allows the detection of other types of variants, especially copy number variations (CNVs), as well as the detection of non-coding variants in regulatory and intronic regions. This has led to an increased diagnostic yield of WGS compared to WES in some studies^13^, which was the highest, especially for the trio-based WGS approach^14^; however, it remains unclear how this will be affected by the selection criteria for undiagnosed patients and what the implications will be in terms of additional costs, increased workload and prolonged turnaround time of results.

In our prospective, single-center study, we have launched a pilot program for pediatric patients with undiagnosed diseases in the second-largest university hospital in the Czech Republic, with WES implemented as a first-line test after enrolment in the study as part of the diagnostic workflow. The aim was to evaluate the diagnostic yield and clinical utility of WES in the Czech population of patients with undiagnosed disease.

## Methods

### Study subjects

This study was prospectively conducted at the Department of Pediatrics at University Hospital Brno (Brno, Czech Republic) between 2020 and 2023. A total of 58 consecutive Czech patients with undiagnosed diseases were enrolled in the study. Exclusion and inclusion criteria were adapted from those used in the Japanese Initiative on Rare and Undiagnosed Diseases (IRUD)^15^, which defines a typical cohort remaining undiagnosed after comprehensive clinical assessment. Patients without highly suspected or known disease, who had not previously undergone WES or WGS and fulfilled the following criteria were eligible for enrollment in this study: (1) the patient remains undiagnosed for 6 months or longer (not necessary for infants), and the symptom(s) affects his/her daily life, AND (2.1) there exists an objective sign(s) that cannot be traced to a single organ, OR (2.2) There is a distinct family history suggestive of a genetic etiology (similar symptom(s) found in the patient’s relatives). To ensure the accuracy of diagnosis, all clinical information and patient records provided by the referring physicians were evaluated carefully. We further applied the human phenotype ontology (HPO) classification to describe the patient’s phenotypes systematically. The study was approved by the University Hospital Brno Ethics Committee (Approvals no. 31-270420 and 12-071222), and informed consent was obtained from all participants or their legal representatives. All methods and analyses were carried out following relevant guidelines and regulations and conducted in accordance with ethical principles and the Code of Ethics of the World Medical Association (Declaration of Helsinki).

### Whole-exome sequencing

DNA was isolated from peripheral blood leukocytes using the QIAmp DNA Micro Kit (Qiagen, Germany). The quantity and purity of the isolated DNA were determined on a NanoDrop 2000c Spectrophotometer and a Qubit 2.0 Fluorometer (ThermoFisher Scientific, MA, USA). Sequencing libraries were prepared using either the TruSeq DNA Exome kit (Illumina, CA, USA) or the KAPA HyperPlus Kit combined with the KAPA HyperExome panel (Roche, Switzerland). Sequencing was performed using the NextSeq 500/550 Mid Output Kit v2.5 (150 cycles) on the NextSeq 500 platform (Illumina, CA, USA) in paired-end setup and read lengths of 2 × 75 bp. More than 97% of the target regions showed at least 40-fold coverage.

### Data analysis, variant detection and classification

Obtained reads were aligned to the GRCh37 (for libraries prepared with TrueSeq DNA Exome kit) or GRCh38 (for libraries prepared using KAPA HyperExome panel) reference genome using the BWA-MEM algorithm (http://bio-bwa.sourceforge.net/) with default parameters. PCR duplicates were removed using Picard tools (http://broadinstitute.github.io/picard/). The aligned BAM files were sorted and extracted using the statistical metric by SAMtools (v.1.9)^16^. A union approach integrating three variant callers, GATK HaplotypCaller (v.3.8)^17^, VarDict^18^, and Strelka^19^, was subsequently used for variant calling. Identified variants were filtered so that only non-synonymous single nucleotide substitutions, insertions, and deletions located in coding and adjacent non-coding (splicing) regions showing a population frequency < 1% according to the GnomAD database (https://gnomad.broadinstitute.org/) were evaluated. Verification was performed using Integrative Genomics Viewer (IGV) software^20^. The potential pathogenicity of the identified variants was evaluated with respect to the impact at the protein level, the distinct biochemical properties of the altered codon, the degree of evolutionary conservation, and also using information available in clinical databases of genetic variants, including OMIM (https://www.omim.org), ClinVar (https://www.ncbi.nlm.nih.gov/clinvar/), or HGMD database (https://www.hgmd.cf.ac.uk/ac/index.php), in population databases (GnomAD and 1000 Genomes, http://www.1000genomes.org/) and in the primary literature. The significance of previously undescribed variants was evaluated using in silico prediction algorithms, such as PolyPhen-2 (http://genetics.bwh.harvard.edu/pph2/), SIFT (https://sift.bii.a-star.edu.sg), Mutation Taster (https://www.mutationtaster.org), or other algorithms integrated into Alamut (https://www.sophiagenetics.com/platform/alamut-visual-plus/) or Varsome tools (https://varsome.com).

Variants were classified according to ACMG recommendations^21^ into one of 5 classes (benign, likely benign, variant of uncertain significance - VUS, likely pathogenic, pathogenic). Pathogenic and likely pathogenic variants in syndrome-associated genes corresponding to the observed proband phenotype and the indication for testing were primarily reported. In addition, variants of uncertain significance present in the zygosity corresponding to the mode of inheritance of the associated syndrome whose significance was supported by prediction algorithms and/or whose manifestation matches the proband phenotype were considered. Beyond the indication for testing, pathogenic and likely pathogenic variants in the panel of 78 genes were evaluated as potential incidental findings in accordance with the ACMG SF 3.1 list^22^.

Selected variants identified by WES were confirmed by Sanger sequencing. A subset of the identified variants was analyzed using Sanger sequencing of the available family members for segregation analysis. For CNV analysis, candidate genes were visually inspected using the IGV software^20,23^, and identified CNVs were confirmed by multiplex ligation-dependent probe amplification. The analysis of the mitochondrial DNA or the WES analysis in the trio was not performed in any of the enrolled patients.

### Interpretation of variants in the clinical context

Identified variants were interpreted considering the patient’s clinical evaluation to assess causality. The proband’s clinical information was transformed to its corresponding standardized HPO terms and assessed to determine the similarity with approximately 7000 rare genetic diseases^24,25^. Variant classification and its predicted functional impact, segregation analysis, and matching with the gene-associated phenotypes known from the literature were considered. A genetic diagnosis was assumed in patients with pathogenic/ likely pathogenic variants and rarely also variants of uncertain significance or their combinations matching the phenotype known from the literature, and these variants were defined as “diagnostic.” The remaining patients were considered undiagnosed^26^.

## Results

### Characteristics of the patients

In the period 1/2020–6/2023 - a total of 58 Czech patients (27 females, 31 males) with a median age of five years (range ten days to 38 years) were enrolled in the study at the Department of Pediatrics at University Hospital Brno, Czech Republic. In total, 55 patients (95%) were aged < 18 years. Only three adult patients (25, 27, and 38 years) followed up from the pediatric age as undiagnosed in the Outpatient Clinic for Inherited Metabolic Disorders at our Department were enrolled. No genetic testing was performed before WES in 28 (48%) patients. In the remaining patients, CMA or targeted genetic testing was performed. Most patients (*n* = 53, 91%) were referred directly from the Department of Pediatrics, four patients (7%) were referred from the Department of Pediatric Neurology, and one patient (2%) from the Department of Pediatric Oncology of our hospital. With one exception described below, the patients were unrelated. There were no consanguineous families in our cohort. When dividing patients into groups, based on the major HPO symptoms at the time of the request for WES, abnormality of metabolism/homeostasis (HP:0001939) were the most frequently observed, followed by a global developmental delay (HP:0001263), dysmorphic facial features (HP:0001999), muscular hypotonia (HP:0001252), failure to thrive (HP:0001508), short stature (HP:0004322) and intellectual disability (HP:0001249) (summarized in Table 1). Three cases in our cohort were published in the form of detailed case reports with the respective references provided. Two patients died before or shortly after WES, at three months^27^ and 2 years of age. In two cases (sisters), WES was performed post-mortem to elucidate the cause of death associated with multiorgan developmental impairment^28^.

**Table 1:** Characteristics of the patients.

Characteristics	*N* (%)
Age at analysis, years, median (range)	5 (0–38)
Sex, n (%)	
Male	31 (53%)
Female	27 (47%)
Major symptoms (HPO*), n	
Global developmental delay (HP:0001263)	19 (33%)
Seizures (HP:0001250)	10 (17%)
Intellectual disability (HP:0001249)	11 (19%)
Muscular hypotonia (HP:0001252)	13 (22%)
Dysmorphic facial features (HP:0001999)	19 (33%)
Short stature (HP:0004322)	11 (19%)
Microcephaly (HP:0000252)	7 (12%)
Macrocephaly (HP:0000256)	3 (5%)
Hepatomegaly (HP:0002240)	7 (12%)
Failure to thrive (HP:0001508)	13 (22%)
Abnormality of metabolism/homeostasis (HP:0001939)	29 (49%)
Abnormality of the eye (HP:0000478)	7 (12%)
Others (congenital malformation syndromes, other neuromuscular symptoms, abnormal behavior)	34 (59%)

*HPO, human phenotype ontology, one patient can have more than one phenotype

### Patients with identified diagnostic variants

The detailed clinical characteristics of the 25 patients with a positive diagnosis are given in Table 2, with a summary below. According to ACMG guidelines^21^, 28 variants, including 11 pathogenic, 13 likely pathogenic, and 4 VUS, were identified as diagnostic of genetic disorders in 25 patients (Table 2). Inheritance patterns of the 25 patients identified as having causal variants were classified as autosomal dominant (*n* = 14; 56%), autosomal recessive (*n* = 10; 40%), or X-linked (*n* = 1; 4%). Eleven variants were confirmed to be *de novo*. The diagnostic variant was inherited from the symptomatic undiagnosed mother in one autosomal dominant case (Table 2; Case 16). The identical *de novo* event in siblings with healthy, variant-negative parents suggests gonadal mosaicism, which can mimic recessive inheritance (Table 2; Cases 7,8)^28^. Four patients had homozygous autosomal recessive variants, and five were compound heterozygotes. In addition, in one case, an X-linked recessive variant was inherited from an asymptomatic mother. Sanger sequencing was performed on 23 patients (92%) and their family members for segregation analysis, with the exception of cases 24 and 25 (Table 2). A total of 26 variants identified by WES were successfully confirmed by Sanger sequencing, including their zygosity. MLPA was successfully used to validate the WES-identified homozygous deletion of the NPHP1 gene (case 25), but the parents’ DNA was unavailable for testing. Eleven novel variants identified in this study had not been previously reported in any public database or scientific literature, including four missense SNVs, three splicing variants, three single-nucleotide deletions, one nonsense SNV, and one large deletion (these variants are bolded in Table 2). We have already published two of them in case reports^27,28^. In our cohort, no pathogenic variants were detected in genes included in the ACMG recommendations for reporting incidental findings^22^.

**Table 2:** Summary of disease-causing variants detected by whole-exome sequencing, phenotypes, diagnosis, and clinical utility of findings.

ID	Age (years)	Sex	Gene (transcript) Variant c., p	ACMG classification	Zygosity	Inheritance	Variant origin	Phenotypes (HPO, Human Phenotype Ontology)	OMIM	Clinical utilitity*, Ref.**
1	12	M	***ALDOB*** (NM_000035.4) c.448G>C, p.A150P	Class 5	Hom	AR	Maternal Paternal	Hepatomegaly (HP:0002240), recurrent hypoglycemia (HP: 0001988)	Hereditary fructose intolerance (#229600)	L, S
2	25	F	***PPP2R5D*** (NM_006245.4) c.592G>A, p.E198K	Class 5	Het	AD	De novo	Global developmental delay (HP: 0001263), Intellectual disability (HP:0001249), delay speech and language development (HP:0000750), macrocephaly (HP:0000256), central hypotonia (HP:0011398), low-set ears (HP:0000369), frontal bossing (HP: 0002007), strabismus (HP: 0000486), dysmorphic facial features (HP:0001999), seizures (HP: 0001250)	Houge-Janssens syndrome 1 (#616355)	R^29^,
3	3 months***	M	***PMM2*** (NM_000303.3) c.691G>A, p.V231M **c.447+3dup**	Class 5 Class 4	Comp. het	AR	Paternal Maternal	Global developmental delay (HP: 0001263), strabismus (HP:0000486), dysmorphic facial features (HP:0001999), inverted nipples (HP: 0003186), coagulopathy (HP:0003256), elevated hepatic transaminases (HP:0002910), failure to thrive (HP:0001508), pericardial effusion (HP:0001698), abnormal fat distribution	Congenital disorder of glycosylation, type Ia (#212065)	None^27^,
4	13	F	***EXT1*** (NM_000127.3) **c.1056+1_1056+2dup**	Class 4	Het	AD	De novo	Multiple exostoses (HP:0002762), short stature (HP:0004322), diabetes mellitus type I (HP:0100651)	Exostoses, multiple, type 1 (#133700)	None
5	14 days	F	***KCNQ2*** (NM_172107.4) c.1678C>T, p.R560W	Class 5	Het	AD	De novo	Focal clonic seizures (HP:0002266), central hypotonia (HP:0011398), foot polydactyly (HP:0001829), hyporeflexia (HP:0001265)	Developmental and epileptic encephalopathy 7 (#613720)	R, M
6	2***	F	***MPZ*** (NM_000530.8) **c.640del, p.R214fs**	Class 4	Het	AD	De novo	Central hypotonia (HP:0011398), hyporeflexia (HP:0001265), fatigable weakness of skeletal muscles (HP:0030196), inverted nipples (HP: 0003186), abnormality of cerebellum (HP:0001317)	Hypomyelinating neuropathy, congenital, 2 (#618184)	None
7	10 days	F	***MYRF*** (NM_001127392.3) **c.1388+2T>G**	Class 4	Het	AD	De novo, germinal mozaicism?	Dyspnea (HP:0002094), hyposaturation, pulmonary hypoplasia (HP:0002089), abnormal bronchial branching (HP:0002109), partial anomalous pulmonary venous return (HP:0010773), pulmonary hypertension (HP:0002092), patent ductus arteriosus (HP:0001643)	Cardiac-urogenital syndrome (#618280)	Post-mortem^28^
8	5 months	F	***MYRF*** (NM_001127392.3) **c.1388+2T>G**	Class 4	Het	AD	De novo, germinal mosaicism?	Dyspnea (HP:0002094), hyposaturation, pulmonary hypoplasia (HP:0002089), abnormal bronchial branching (HP:0002109), partial anomalous pulmonary venous return (HP:0010773), pulmonary hypertension (HP:0002092), coarctation of aorta (HP:0001680), hypoplastic aortic arch (HP:0012304), patent ductus arteriosus (HP:0001643)	Cardiac-urogenital syndrome (#618280)	Post-mortem^28^
9	3	F	***DYRK1A*** (NM_001347721.2) c.586C>T, p.R196*	Class 5	Het	AD	De novo	Global developmental delay (HP:0001263), intellectual disability (HP:0001249), feeding difficulties (HP:0011968), failure to thrive (HP:0001508), microcephaly (HP:0000252), lower limb spasticity (HP:0002061), dysmorphic facial features (HP:0001999)	Intellectual developmental disorder, autosomal dominant 7 (#614104)	R, S
10	12	M	***PHKA2*** (NM_000292.3) c.270C>T, p.R824C	Class 4	Hemizyg	XLR	Maternal	Hepatomegaly (HP:0002240), recurrent mild hypoglycemia (HP: 0001988), elevated hepatic transaminases (HP:0002910), failure to thrive (HP:0001508), hyperlipidemia (HP:0003077)	Glycogen storage disease, type Ixa (#306000)	L, S
11	17	F	***ABCC8*** (NM_000352.6) **c.4104C>G, p.I1368M** c.4008G>C, p.K1336N	Class 4 Class 3	Comp. het	AR	Paternal Maternal	Hypoglycemia (HP:0001943), seizures (HP:0001250)	Hyperinsulinemic hypoglycemia, familial, 1 (#256450)	R, M, S
12	6	F	***SGSH*** (NM_000199.5) c.1167C>A, p.N389K **c.1483T>C, p.C495R**	Class 5 Class 4	Comp. het	AR	Paternal Maternal	Global developmental delay (HP: 0001263), Intellectual disability (HP:0001249), delay speech and language development (HP:0000750), abnormal emotion/affect behaviour (HP: 0100851), dysmorphic facial features (HP:0001999), coarse facial features (HP:0000280), abnormality of hair texture (HP:010719), short stature (HP:0004322)	Mucopolysaccharidosis type IIIA (Sanfilippo A) (#252900)	R
13	5	F	***FBXO11*** (NM_001190274.2) c.1810C>T, p.Q604*	Class 4	Het	AD	De novo	Microcephaly (HP:0000252), myopathy (HP:0003198), global developmental delay (HP:0001263), joint hypermobility (HP:0001382), intellectual disability (HP:0001249), dysmorphic facial features (HP:0001999)	Intellectual developmental disorder with dysmorphic facies and behavioral abnormalities (#607871)	None
14	14	M	***MEGF10*** (NM_001256545.2) **c.1562G>C, p.R521P**	Class 3	Hom	AR	Maternal Paternal	Failure to thrive (HP:0001508), fatigable weakness of skeletal muscles (HP:0030196), myopathy (HP:0003198), scoliosis (HP:0002650), high palate (HP:0000218), areflexia (HP:0001284), hypoventilation (HP: 0002791), hypercapnia (HP:0002791)	Congenital myopathy 10B, mild variant (#620249)	R, Su, P, I, M
15	4	M	***PYGL*** (NM_002863.4) c.2017G>A, p.E673K	Class 4	Hom	AR	Maternal Paternal	Hepatomegaly (HP:0002240), elevated hepatic transaminases (HP:0002910), failure to thrive (HP:0001508), hyperlipidemia (HP:0003077), Postnatal growth retardation (HP:0008897), short stature (HP:0004322)	Glycogen storage disease VI (#613741)	L, S, R
16	1	F	***TRIO*** (NM_007118.4) **c.2388del, p.I796fs**	Class 4	Het	AD	Maternal	Microcephaly (HP:0000252), global developmental delay (HP:0001263), dysmorphic facial features (HP:0,001,999), brachycephaly (HP:0000248), premature closure of fontanelles (HP: 0005458), epicantus (HP:0000286), frontal bossing (HP: 0002007), wide nose (HP:0000445)	Intellectual developmental disorder, autosomal dominant 44, with microcephaly (#618825)	None
17	3	M	***NSD1*** (NM_022455.5) c.5127G>A, p.W1709*	Class 5	Het	AD	De novo	Macrocephaly (HP:0000256), tall stature (HP:0000098), neuroblastoma (HP:0003006), dysmorphic facial features (HP:0001999), deeply set eye (HP:0000490), low-set ears (HP:0000369), frontal bossing (HP: 0002007), elevated hepatic transaminases (HP:0002910), intellectual disability (HP:0001249)	Sotos syndrome (#117550)	S
18	1 month	M	***RARS2*** (NM_020320.5) c.1679G>A, p.R560H **c.1157G>A, p.R386Q**	Class 4 Class 3	Comp. het	AR	Paternal Maternal	Microcephaly (HP:0000252), global developmental delay (HP:0001263), seizures (HP:0001250), central hypotonia (HP:0011398), feeding difficulties (HP:0011968), high lactate level, dysmorphic facial features (HP:0001999)	Pontocerebellar hypoplasia, type 6 (#611523)	S, M, I
19	1	F	***GBE1*** (NM_000158.4) c.691+2T>C **c.113T>G, p.L38W**	Class 5 Class 3	Comp. het	AR	Paternal Maternal	Hepatomegaly (HP:0002240), elevated hepatic transaminases (HP:0002910), failure to thrive (HP:0001508), central hypotonia (HP:0011398), global developmental delay (HP: 0001263), short stature (HP:0004322)	Glycogen storage disease IV (#232500)	R, L, S
20	1 month	F	***GRIN2B*** (NM_000834.5) c.2065G>A, p.G689S	Class 5	Het	AD	De novo	Dysmorphic facial features (HP:0001999), global developmental delay (HP: 0001263), central hypotonia (HP:0011398), short stature (HP:0004322), failure to thrive (HP:0001508), feeding difficulties (HP:0011968), strabismus (HP:0000486)	Intellectual developmental disorder, autosomal dominant 6, with or without seizures (#613970)	R, S
21	9	F	***GNAO1*** (NM_020988.3) c.625C>T, p.R209C	Class 5	Het	AD	De novo	Seizures (HP:0001250), global developmental delay (HP: 0001263), intellectual disability (HP:0,001,249), central hypotonia (HP:0011398), failure to thrive (HP:0001508), dystonia (HP:0001332), dyskinesias (HP: 0100660), spastic tetraparesis (HP:0001285)	Neurodevelop—mental disorder with involuntary movements (#617493)	P, S
22	3 weeks	M	***G6PC1*** (NM_000151.4) c.247C>T, p.R83C	Class 5	Hom	AR	Paternal Maternal	Fasting hypoglycemia (HP:0003162), hepatomegaly (HP:0002240), elevated hepatic transaminases (HP:0002910), lactic acidosis (HP:0003128)	Glycogen storage disease Ia (#232200)	R, S, L, P
23	6 weeks	F	***KMT2D*** (NM_003482.4) c.5124_5125del, p.R1709fs	Class 5	Het	AD	De novo	Seizures (HP:0001250), hypoglycemia (HP: 0001943), Mondini malformation HP:0000376, dysmorphic facial features (HP:0001999), wide nose (HP:0000445), hypertelorism (HP:0000316), long palpebral fissures (HP:0000637), blue sclerae (HP:0000592), iris coloboma (HP:0000612), suspect visual impairment (HP:0000505)	Kabuki syndrome 1 (#147920)	R, S
24	14	M	***NFIB*** (NM_001190737.2)** c.701del, p.G234fs**	Class 4	Het	AD	Unknown	Global developmental delay (HP: 0001263), Intellectual disability (HP:0001249), delayed speech and language development (HP:0000750), macrocephaly (HP:0000256), strabismus (HP: 0000486), abnormal emotion/affect behaviour (HP: 0100851), dysmorphic facial features (HP:0001999), coarse facial features (HP:0000280), thick eyebrown (HP:0000574), short stature (HP:0004322)	Macrocephaly, acquired, with impaired intellectual development (#618286)	none
25	8	M	***NPHP1*** deletion exon 1–20	Pathogenic	Hom	AR	Unknown	Amblyopia (HP:0000646), astigmatism (HP: 0000529), visual impairment (HP:0000505), renal insuficiency (HP:0000083)	Senior-Loken syndrome-1 (#266900)	P, R, S

*Clinical utility is defined as a change in the clinical management following a causative diagnosis by WES, including, but not limited to, surveillance (S), referral to specialists (R), and indication or contraindication of investigations (I), procedures (P), surgeries (Su), medications (M) and lifestyle change (L). Genetic counseling and reproductive planning were not included in clinical utility because they were assumed to apply to all diagnostic tests.

**Ref., reference to publication if already published as a case report.

*** Patient died before or shortly after WES.

Bolded variants are the novel variants identified in this study that had not been previously reported in any public database.

### Diagnostic yield and clinical utility

Of the 58 probands, 28 disease-causing variants were detected in 25 individuals, resulting in an overall diagnostic yield of 43%. The clinical utility (actionability) or the way WES-based diagnosis can affect medical care or the life of the patient could be classified in various ways, but definitely, it should enable a more personalized approach to clinical management. Implications for patient care in our patient cohort were as follows (summarized in Table 2): change in surveillance (*n* = 15), referral to specialists (*n* = 14), indication or contraindication of investigations (*n* = 6), procedures (*n* = 4), surgeries (*n* = 1), medication change (*n* = 2) and lifestyle change (e.g. dietary recommendations, *n* = 5). The overall clinical utility enabling at least one type of actionability was 76% (19 patients), whereas the average number of clinical implications to individual patient care was two. If we exclude the two most frequent types of actionability, i.e., referral and surveillance, the medical care or patient lifestyle will be affected in 11 patients (44%). In only 6 (24%) out of 25 children, the WES-based diagnosis did not allow any further intervention and did not affect patient care.

## Discussion

The “diagnostic odyssey” is a well-known term in the field of rare and ultra-rare diseases. Undiagnosed conditions include those with rare, difficult-to-identify conditions, atypical presentations of known conditions, and diseases yet to be discovered^1^. Patients and their families can spend years on this diagnostic odyssey and never arrive at a diagnosis. The average length of a diagnostic odyssey is eight years^2^. Therefore, undiagnosed disease programs and networks have emerged to address the significant number of these individuals with suspected but undiagnosed rare genetic diseases to optimize their diagnostic and clinical outcomes.

Diagnostic opportunities for these patients were markedly shifted with the advent of genome-wide sequencing tests, like WES or WGS. Comparing WES and WGS, there is evidence showing the capability of WGS to achieve molecular diagnoses for cases that remain undiagnosed after WES^30,31^. For example, Wu et al. compared the diagnostic yield of WES and WGS and showed that WES missed 10 of 74 (14%) diagnoses because of disease-causing deep intronic, non-coding variants, and structural variants^31^. On the other hand, the diagnostic rate of WGS did not differ significantly from that of WES in the two meta-analyses^12,32^. Therefore, considering the cost-benefit ratio, increased workload, and prolonged turnaround time of results in the case of WGS, we decided to implement WES for our study.

Currently, based on the ACMG recommendations^33^, genome-wide sequencing tests should be performed as first- or at least second-tier test in undiagnosed patients with a suspected genetic condition. Although the efficacy of this approach is widely demonstrated in the literature, these guidelines are far from being applied in real-world settings, including the common practice in the Czech Republic.

In the present study, WES was implemented as the first genetic test after inclusion in the study as part of the diagnostic workflow in the cohort of 58 Czech pediatric patients with undiagnosed disease. Regarding ACMG recommendations, 48% of patients in our cohort had no previous genetic testing performed, and WES presented a first-tier test. In the remaining patients, it was a second-tier genetic test after CMA or targeting gene testing. All patients fulfilled the criteria of the Japanese Initiative on Rare and Undiagnosed Diseases (IRUD), which we adapted for our study. The IRUD criteria differ from those used by the US UDN network because they do not specify a minimum number of specialist examinations a patient must undergo to be enrolled in the program^14^.

Overall diagnostic yield and clinical utility achieved in our cohort were 43% and 76%, respectively. This diagnostic yield is consistent with the one calculated in the recently published meta-analysis, where the median diagnostic rate of WES in a subset of 13 high-quality studies (as assessed by QUADAS-2)^34^ was 43% (95% CI 0.35–0.51, *n* = 2612)^12^. In the same meta-analysis, the median clinical utility of WES observed in the four available high-quality studies was 44% (95% CI 0.30–0.58, *n* = 723)^12^, which is much lower than the one observed in our study. However, meta-analysis also shows that the clinical utility of WES ranges from 2 to 100% in the diagnosed patients when all 62 evaluated cohorts are included. Such a wide range of clinical utility rates across studies can be explained by the inconsistent definitions used for clinical utility and its categories in the various studies. Here, we used the most common classification of clinical utility into the following categories: changes in surveillance, referral to specialists, lifestyle changes and diet, indication or contraindication of investigations, procedures, surgeries, and medication changes.

Sanger sequencing is recommended to be used for verification of WES-findings to avoid false-positive variant calls^26^. In our study, all 28 diagnostic variants were successfully validated by Sanger sequencing. Family member testing by Sanger sequencing and segregation analysis was performed in 23 (92%) out of 25 patients with identified diagnostic variants, which enabled clarification of the inheritance pattern and carrier status in family members. When using WES, copy number variations (CNVs) can be identified by analyzing read depth and coverage across exon regions. However, the accuracy of CNVs estimation in targeted or exome sequencing is constrained by the considerable variability in sequencing coverage and read depth across different regions^23^. Therefore, the only gene deletion (case 25) identified by WES in our cohort was successfully validated by MLPA.

Our study has several limitations. First, the small number of patients enrolled did not allow statistical evaluation of the diagnostic yield and clinical utility of singleton WES in various subgroups of patients stratified by major symptoms. Further, the use of trio WES may increase diagnostic yields compared with singleton WES, especially when we consider the potential diagnostic variants of uncertain significance. Despite its advantages, WES also has some of the aforementioned limitations regarding the types of variants that can be detected. Some of these limitations may explain why 57% of our cohort failed to obtain a genetic diagnosis even after WES. In some of the undiagnosed cases, trio WES, WGS, or trio WGS could probably provide a molecular diagnosis.

## Conclusion

In conclusion, our study provides evidence, for the first time in the Czech population, that genome-wide sequencing tests, such as WES, enable high diagnostic yield and clinical utility in pediatric patients with undiagnosed diseases. In our cohort, we achieved the upper limits of diagnostic yields and clinical utility published in analogous studies from other countries where WES was applied in undiagnosed pediatric patients. Of the 28 diagnostic variants identified, twelve were novel and had not been previously reported in any public database.

## Data Availability

Sequence data has been deposited at the European Genome-phenome Archive (EGA), under accession number EGAS50000000442.
